# Incident low muscle mass is associated with greater lung disease and lower circulating leptin in a tobacco-exposed longitudinal cohort

**DOI:** 10.1186/s12931-023-02521-3

**Published:** 2023-09-22

**Authors:** Richard H. Zou, S. Mehdi Nouraie, Chad Karoleski, Yingze Zhang, Frank C. Sciurba, Daniel E. Forman, Jessica Bon

**Affiliations:** 1 Division of Pulmonary, Allergy, and Critical Care Medicine, Department of Medicine, University of Pittsburgh Medical Center, Pittsburgh, PA USA; 2 Emphysema COPD Research Center, University of Pittsburgh, Pittsburgh, PA USA; 3 Division of Cardiology, Department of Medicine, University of Pittsburgh Medical Center, Pittsburgh, PA USA; 4 Division of Geriatrics, Department of Medicine, University of Pittsburgh Medical Center, Pittsburgh, PA USA; 5 Veteran Affairs Pittsburgh Healthcare System, Pittsburgh, PA USA; 6 UPMC Montefiore Hospital, NW628 3459 Fifth Avenue, Pittsburgh, PA 15213 USA

**Keywords:** Pulmonary disease, Chronic obstructive, Sarcopenia, Adipokines

## Abstract

**Background:**

Muscle loss is prevalent in chronic obstructive pulmonary disease (COPD). Prior studies evaluating musculoskeletal dysfunction in COPD have focused on individuals with baseline low muscle mass. Currently, there is limited data evaluating clinical characteristics and outcomes associated with progression to incident low muscle mass in a tobacco-exposed cohort of individuals with baseline normal muscle mass.

**Methods:**

We evaluated 246 participants from a single-center longitudinal tobacco-exposed cohort with serial spirometry, thoracic imaging, dual energy x-ray absorptiometry (DXA) measurements, walk testing, and plasma adipokine measurements. DXA-derived fat free mass index (FFMI) and appendicular skeletal mass index (ASMI) were used as surrogates for muscle mass. Participants with incident low muscle mass (LM) at follow-up were characterized by FFMI < 18.4 kg/m^2^ in males and < 15.4 kg/m^2^ in females and/or ASMI < 7.25 kg/m^2^ in males and < 5.67 kg/m^2^ in females.

**Results:**

Twenty-five (10%) participants progressed to incident low muscle mass at follow-up. At baseline, the LM subgroup had greater active smoking prevalence (60% v. 38%, *p* = 0.04), lower FFMI (17.8 ± 1.7 kg/m^2^ v. 19.7 ± 2.9 kg/m^2^, *p* = 0.002), lower ASMI (7.3 ± 0.9 kg/m^2^ v. 8.2 ± 1.2 kg/m^2^, *p* = 0.0003), and lower plasma leptin (14.9 ± 10.1 ng/mL v. 24.0 ± 20.9 ng/mL, *p* = 0.04). At follow-up, the LM subgroup had higher COPD prevalence (68% v. 43%, *p* = 0.02), lower FEV_1_/FVC (0.63 ± 0.12 v. 0.69 ± 0.12, *p* = 0.02), lower %DLco (66.5 ± 15.9% v. 73.9 ± 16.8%, *p* = 0.03), and higher annual rate of FFMI decline (-0.17 kg/m^2^/year v. -0.04 kg/m^2^/year, *p* = 0.006). There were no differences in age, gender distribution, pack years smoking history, or walk distance.

**Conclusions:**

We identified a subgroup of tobacco-exposed individuals with normal baseline muscle mass who progressed to incident DXA-derived low muscle mass. This subgroup demonstrated synchronous lung disease and persistently low circulating leptin levels. Our study suggests the importance of assessing for muscle loss in conjunction with lung function decline when evaluating individuals with tobacco exposure.

**Supplementary Information:**

The online version contains supplementary material available at 10.1186/s12931-023-02521-3.

## Background

Chronic obstructive pulmonary disease (COPD) is a heterogeneous chronic lung disease with multisystemic manifestations that contribute significantly to disease morbidity and mortality [1, 2]. Decreased muscle mass is an important extrapulmonary manifestation of COPD, with estimated prevalence ranges between 15 and 40% [3]. In individuals with COPD, reduced muscle mass is associated with lung disease progression, exercise intolerance, decreased health-related quality of life, and mortality [4–8]. In recent years, physical inactivity and frailty have become growing areas of focus in this high-risk population [9]. Dysregulated adipokine metabolism – with adiponectin, leptin, and resistin being commonly implicated adipokines – is also prevalent in COPD and is associated with inflammatory response regulation, emphysema, and skeletal muscle dysfunction [10–13].

Body mass index (BMI) is commonly used for risk stratification in COPD but may not accurately reflect true muscle mass, as pathologic loss of skeletal muscle is not always accompanied by proportional loss of adipose tissue [14]. Dual energy x-ray absorptiometry (DXA) is the gold standard for body composition measurement in COPD [15–17]. Fat free mass index (FFMI), defined as the sum of whole-body lean muscle and bone mineral content adjusted for height in meters squared, is a clinically accepted and widely used surrogate marker for muscle mass in COPD [18, 19]. In cross sectional COPD studies, FFMI correlates with airflow obstruction and emphysema, and is an independent predictor of mortality, regardless of BMI [20, 21]. The dynamic processes of FFMI change over time and longitudinal weight loss are also associated with increased mortality [22, 23]. Similarly, appendicular skeletal mass index (ASMI), defined as the sum of upper and lower extremity lean muscle adjusted for height in meters squared, is another DXA measure that associates with impaired functional status in COPD [24]. However, clinicians do not routinely order whole-body DXA scans to specifically assess for FFMI and/or ASMI in COPD, but rather rely on BMI, despite its limitations, as a global assessment of body composition.

Prior studies evaluating muscle loss in COPD have used clinical cohorts that included individuals with prevalent low muscle mass [18, 19, 21]. Additionally, these studies utilized alternative approaches for defining muscle loss, such as quantitative computed tomography (CT) measurements, [25, 26] which lack established thresholds and are not incorporated into current definitions of sarcopenia [27]. Currently, there are few studies describing risk factors associated with index progression to muscle loss using gold standard DXA measurements of body composition. Longitudinal evaluation of risk factors associated with incident low muscle mass using gold standard definitions is necessary to accurately identify the subgroup at highest risk for disease progression and morbidity who may benefit most from early targeted interventions.

Importantly, most studies describing musculoskeletal comorbidities and outcomes in lung disease have primarily focused on participants with COPD. However, tobacco exposure is associated with oxidative stress in peripheral airways and muscle, and tobacco-exposed individuals without airflow obstruction remain at high risk for functional status decline, muscle dysfunction, and muscle loss [23, 28–30]. Therefore, evaluation and identification of incident low muscle mass should focus on all tobacco-exposed individuals, regardless of spirometry.

The main purposes of this study were to [1] identify clinical characteristics and adipokines associated with incident low muscle mass and [2] evaluate associations between incident low muscle mass and clinical outcomes in a tobacco-exposed longitudinal cohort with and without airflow obstruction.

## Methods

### Study population

**Fig. 1:**
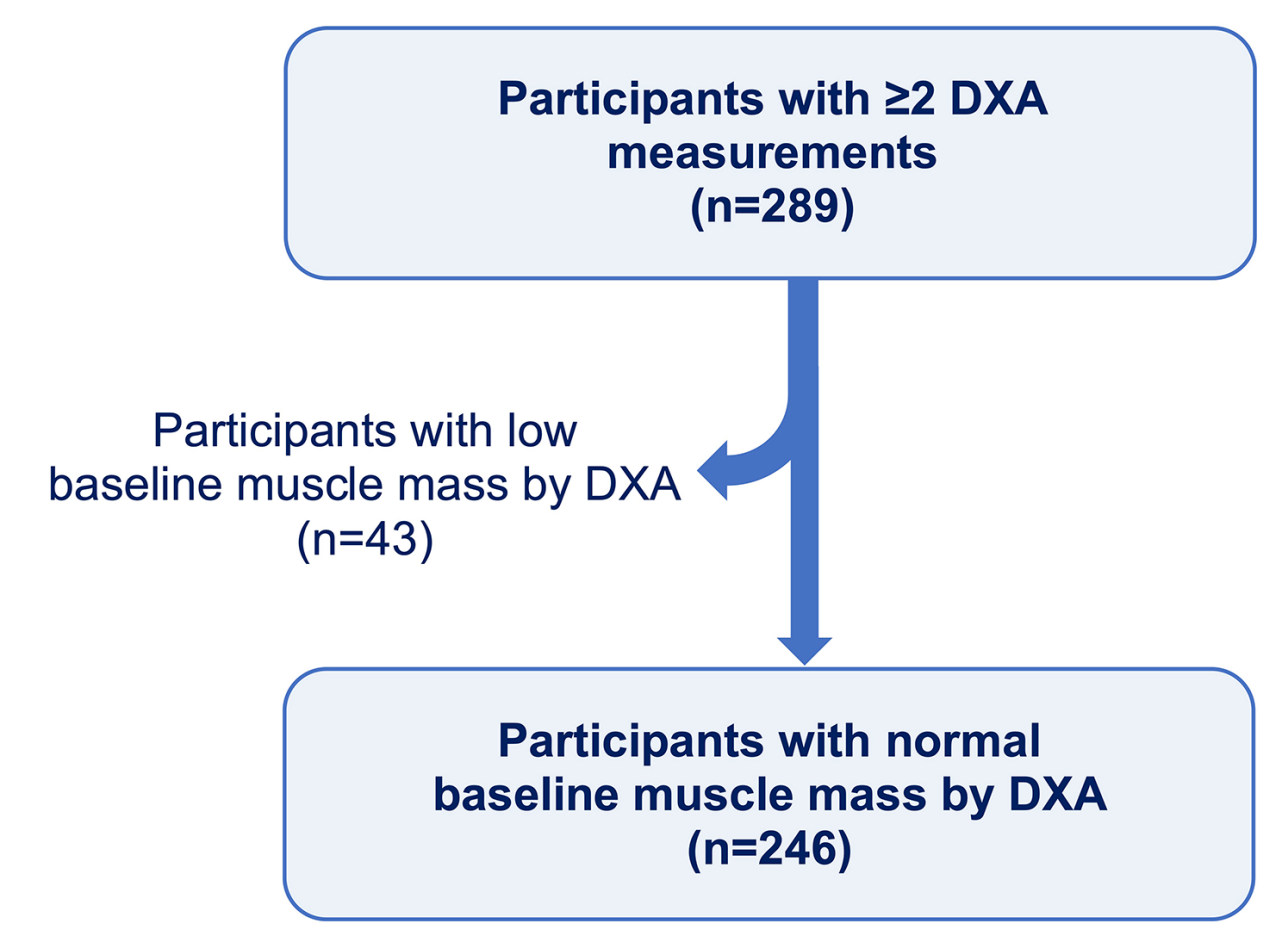
Study flow diagram. Eligible participants were ≥40 years of age, current or former tobacco smoke users, and had ≥10 pack year smoking history. Participants were followed between 2 to 10 years after the baseline study visit

Participants from the single-center Specialized Centers of Clinically Oriented Research (SCCOR) longitudinal cohort at the University of Pittsburgh were enrolled according to the previously published study design [25]. All participants were ≥ 40 years of age at time of enrollment and had ≥ 10 pack year smoking history. Pertinent exclusion criteria included clinical or radiographic evidence of alternative primary lung processes, suspicious lung nodule or lung malignancy, chronic systemic corticosteroid use, and BMI ≥ 36 kg/m^2^. Participants were followed over several study visits, with longitudinal data ranging between 2 and 10 years (median 6 years, IQR 5–8 years). At each study visit, participants completed pulmonary function testing, chest computed tomography (CT) imaging, DXA measurements of body composition, incremental shuttle walk testing (ISWT), respiratory-specific questionnaires, and blood collection. For this study, we used a convenience sample of 246 participants who had normal baseline muscle mass at cohort entry (defined in the *Body Composition Measurement* subsection) and ≥2 DXA measurements (Fig. 1). The University of Pittsburgh Institutional Review Board approved all data acquisition procedures (*IRB CR19090239-006*) and written informed consent was obtained from all participants.

### Body composition measurements

Body mass index (BMI) was defined as weight in kilograms (kg) over height in meters squared (m^2^). Normal BMI range is 18.5–24.9 kg/m^2^ [31]. DXA measures of body composition were obtained using a *Hologic Discovery* densitometer (*Hologic Inc., Bedford, MA, USA*). Fat free mass index (FFMI) was defined as the sum of whole-body lean muscle and bone mineral content in kg over height in m^2^. Low FFMI was defined as < 18.4 kg/m^2^ in males and < 15.4 kg/m^2^ in females based on FFMI < 25th percentile of healthy adults aged 55–74 [32]. Appendicular skeletal mass index (ASMI) was defined as the sum of upper and lower extremity lean muscle in kg over height in m^2^. Low ASMI was defined as < 7.25 kg/m^2^ in males and < 5.67 kg/m^2^ in females based on ASMI < 20th percentile of healthy elderly adults [33].

### Clinical measurements

Pulmonary function testing with pre- and post-bronchodilator challenge and diffusing capacity of the lung for carbon monoxide (DLco) was performed using American Thoracic Society (ATS) standards [34]. COPD was defined by a post-bronchodilator ratio of forced expiratory volume in 1 s (FEV_1_) to forced vital capacity (FVC) < 0.70. The degree of airflow obstruction was characterized by percent predicted FEV_1_ (%FEV_1_) using Gold Initiative for Chronic Obstructive Lung Disease (GOLD) criteria, with mild (stage I), moderate (II), severe (III), and very severe (IV) represented by FEV_1_ ≥ 80%, between ≥ 50% and < 80%, between ≥ 30% and < 50%, and < 30%, respectively [35].

Incremental shuttle walk testing (ISWT) was used to assess maximal performance capacity [36]. Participants walked along a stretch of unimpeded hallway at a pace dictated by a recording on a cassette player with the pace incrementally increased over 12 stages until exercise was stopped due to symptoms. All participants who were prescribed oxygen therapy utilized their supplemental oxygen during testing.

The St. George’s Respiratory Questionnaire (SGRQ), a COPD-specific validated questionnaire for health status assessment, was used to determine health-related quality of life and symptom burden [37]. SGRQ scores range between 0 and 100, where higher scores indicate greater respiratory symptom burden. Subdomains included Activity, Impacts, and Symptoms. The modified Medical Research Council (mMRC) Dyspnea Scale, a validated 5-point self-reported scoring questionnaire, was used to determine dyspnea severity and disability [38]. Severe exacerbations, defined by those requiring hospitalization, were dichotomized as absent or present and self-reported at each study visit. Pulmonary rehabilitation was dichotomized as absent or present and self-reported at each study visit.

### Radiographic assessment

Chest CT scans were performed using a standard high-resolution protocol at 0.625-millimeter thickness and interval. Quantitative emphysema was measured using the 15th percentile voxel point (Perc15), where more negative values (i.e., closer to -1,000 Hounsfield Units) correspond to greater emphysema burden [39]. Semi-quantitative emphysema was visually determined using the Emphysema Score (EScore), a validated 6-point scoring system determined by a single board-certified thoracic radiologist blinded to participant characteristics [40]. EScores defined as absent (score 0), trace/minimal [1], mild [2], moderate [3], severe [4], and very severe [5] correspond to < 10%, 10–25%, 26–50%, 51–75%, and > 75% visual emphysema, respectively.

### Blood adipokine measurement

Plasma levels of adiponectin, leptin, and resistin were measured in the fasting state using the human obesity multiplex Luminex assays (*R&D Systems, Minneapolis, Minnesota, USA*) and analyzed using a Bioplex 200 platform (*Bio-Rad, Hercules, California, USA*) from banked participant samples collected at baseline and final follow-up study visits. All samples were run in duplicates and analyzed according to the manufacturer’s instructions.

### Statistical analysis

Participants were grouped based on the presence or absence of incident low muscle mass at their final follow-up study visit, defined by low FFMI and/or ASMI. Differences in baseline and follow-up clinical, physiologic, radiographic, and adipokine levels between the incident low muscle mass (LM) subgroup and the stable muscle mass (SM) subgroup were assessed using chi-squared test for categorical variables, two-sample t-test for normally distributed baseline variables, and Wilcoxon rank-sum test for non-normally distributed baseline variables. Bivariate and multivariate logistic regression modeling were used to determine relationships between longitudinal muscle phenotypes and plasma adipokine levels. Multivariable partial correlation coefficient modeling was used to determine relationships between plasma adipokine levels and DXA-derived total body fat mass. Receiver operator characteristic (ROC) curve and Youden index were used to determine the area under curve (AUC) and the optimal cut-point of baseline plasma leptin level for discriminating between LM and SM subgroups. Covariates included age, gender (due to muscle and fat tissue differences between males and females), %FEV_1_, active smoking status, and corticosteroid use. Relationships were reported using odds ratios (OR) with 95% confidence intervals or partial Pearson correlation coefficients (*r*) with associated p-values. Kernel density estimates were used to provide non-normally distributed smoothed probability density estimates of the variable of interest (calculated annual rate of FFMI change) in histogram format. Missing data was assumed to be missing at random for the purposes of this longitudinal cohort study. All statistical analyses were performed using Stata 17.1 (*StataCorp, Inc., College Station, Texas, USA*). Figures were created using GraphPad Prism 9.5 (*GraphPad Software, Boston, Massachusetts, USA*).

## Results

### Baseline study cohort characteristics

At baseline, our study cohort had a mean age of 64.6 ± 5.7 years, equal gender distribution (52% male, 48% female), and significant tobacco use (median 44 pack years, interquartile range [IQR] 31–60 pack years) (Table 1). Ninety-nine (40%) participants were active tobacco users at baseline and 80 (33%) remained active tobacco users at follow-up. A total of 150 (61%) participants had lung disease, defined by airflow obstruction and/or emphysema. One hundred two (42%) participants met GOLD criteria for COPD, with the majority demonstrating mild (41%) or moderate (50%) airflow obstruction. Half of participants had CT-determined emphysema, with the majority demonstrating trace/minimal (58%), mild (20%), or moderate (17%) disease. Mean anthropometric measurements were notable for BMI of 29.1 ± 3.5 kg/m^2^ (male: 29.1 ± 3.2 kg/m^2^, female: 29.0 ± 3.8 kg/m^2^), FFMI of 19.6 ± 2.3 kg/m^2^ (male: 21.1 ± 1.7 kg/m^2^, female: 17.8 ± 1.5 kg/m^2^), and ASMI of 8.1 ± 1.2 kg/m^2^ (male: 8.9 ± 0.9 kg/m^2^, female: 7.2 ± 0.7 kg/m^2^). The interval range between first and final DXA measurements was 1.8 to 6.6 years.

**Table 1:** Baseline and final follow-up study cohort characteristics. A total of 246 participants had demographic, clinical, radiographic, and body composition data. Values are listed as mean with standard deviation, unless otherwise specified by *, indicating median with interquartile range. There were no available updated total pack years at final follow-up

	Baseline (*n* = 246)	Final Follow-Up (*n* = 246)
Age (y)	64.6 (5.7)	70.7 (5.8)
Male sex (n, %)	129 (52%)	129 (52%)
Caucasian race (n, %)	230 (93%)	230 (93%)
Pack years *	44 (31–60)	-
Active smoking (n, %)	99 (40%)	80 (33%)
Inhaled corticosteroids (n, %)	23 (9%)	44 (18%)
Oral corticosteroids (n, %)	4 (2%)	4 (2%)
BMI (kg/m^2^)	29.1 (3.5)	28.9 (4.1)
FFMI (kg/m^2^)	19.6 (2.3)	19.2 (2.5)
ASMI (kg/m^2^)	8.1 (1.2)	7.8 (1.2)
Total body fat mass (kg)	27.5 (7.6)	27.8 (8.2)
Spirometry		
FEV_1_/FVC FEV_1_ predicted (%) DLco predicted (%)	0.69 (0.11) 87.7 (18.7) 77.8 (16.1)	0.68 (0.12) 86.5 (21.1) 73.2 (16.8)
COPD (n, %) GOLD I (n, %) GOLD II (n, %) GOLD III (n %) GOLD IV (n, %)	102 (42%) 42 (41%) 51 (50%) 9 (9%) 0 (0%)	113 (46%) 40 (35%) 58 (51%) 13 (12%) 1 (1%)
Emphysema by EScore (n, %) Trace/Minimal (n, %) Mild (n, %) Moderate (n, %) Severe (n, %) Very Severe (n, %)	123 (50%) 71 (58%) 24 (20%) 21 (17%) 4 (3%) 3 (2%)	116 (49%) 58 (50%) 34 (29%) 18 (16%) 3 (3%) 3 (3%)
Perc15 (HU)	-902 (24)	-905 (24)
SGRQ Total score *	12 (6–28)	13 (5–25)
SGRQ Activity score *	19 (6–42)	23 (6–42)
SGRQ Impacts score *	4 (0–14)	4 (0–13)
SGRQ Symptoms score *	23 (10–38)	21 (9–35)
mMRC score *	0 (0–1)	1 (0–1)
Walk distance (m)	456 (143)	371 (142)
Severe exacerbation (n, %)	10 (4%)	9 (4%)
Pulmonary rehabilitation (n, %)	2 (< 1%)	8 (3%)

### Muscle mass change is variable over time

**Fig. 2:**
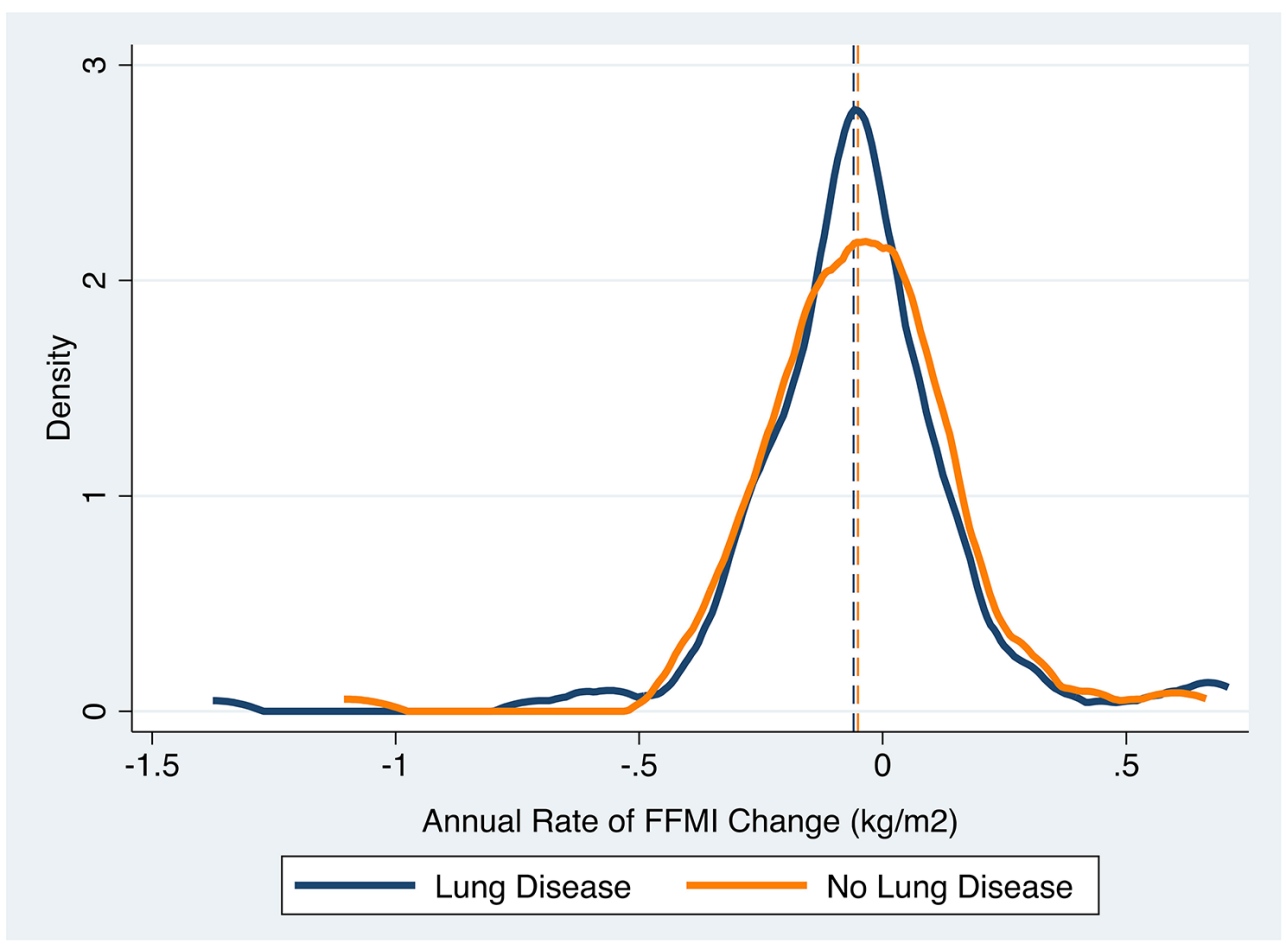
Annual rates of FFMI change are variable and not modified by lung disease. Distribution of annual change of FFMI (x-axis) is plotted against density (y-axis). Subgroup mean values are indicated by vertical lines. Lung disease is defined by airflow obstruction and/or emphysema. ∆: change. Lung disease: -0.06 kg/m^2^/year. No lung disease: -0.05kg/m^2^/year

In longitudinal analyses using kernel density estimate plotting, our tobacco-exposed cohort demonstrated variable annual rates of FFMI change over time (Fig. 2). There were no meaningful differences in the mean annual rate of FFMI change between participants with lung disease (-0.06 kg/m^2^/year) and participants without lung disease (-0.05 kg/m^2^/year).

**Fig. 3:**
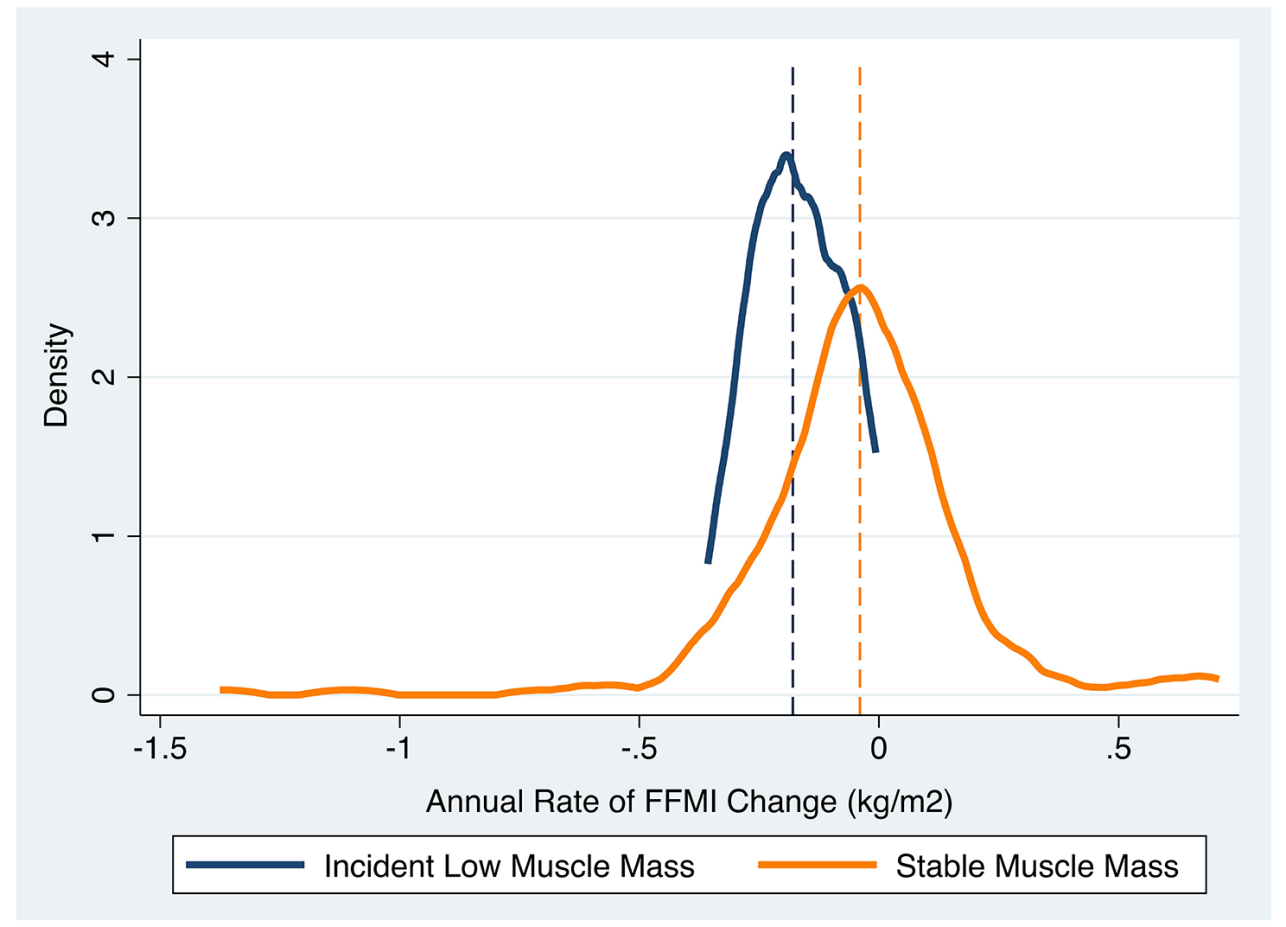
Annual rates of FFMI change is greater for LM subgroup. Distribution of annual change of FFMI (x-axis) is plotted against density (y-axis). Subgroup mean values are indicated by vertical lines. ∆: change. LM subgroup: -0.17 kg/m^2^/year. SM subgroup: -0.04kg/m^2^/year

The incident low muscle mass (LM) subgroup (*n* = 25) had significantly higher annual rate of FFMI decline compared with the stable muscle mass (SM) subgroup (*n* = 221) (-0.17 kg/m^2^/year v. -0.04 kg/m^2^/year, *p* = 0.006) (Fig. 3). No participants in the LM subgroup gained muscle, whereas 85 participants (38%) in the SM subgroup gained muscle. The LM subgroup had a median follow-up period of 7 years (IQR 5–10 years), and the SM subgroup had a median follow-up period of 6 years (IQR 4–7 years) (*p* = 0.01).

### Incident low muscle mass is associated with lower lung function at follow-up

We compared baseline study cohort characteristics between the LM and SM subgroups (Table 2). Participants in the LM subgroup had lower BMI (25.7 ± 2.0 kg/m^2^ v. 29.4 ± 3.4 kg/m^2^, *p* < 0.0001), lower FFMI (17.8 ± 1.7 kg/m^2^ v. 19.7 ± 2.9 kg/m^2^, *p* = 0.002), lower ASMI (7.3 ± 0.9 kg/m^2^ v. 8.2 ± 1.2 kg/m^2^, *p* = 0.0003), and greater active tobacco use (60% v. 38%, *p* = 0.04). There were no differences in age, gender distribution, total pack years, lung function, emphysema, respiratory symptoms, corticosteroid use, walk distance, severe exacerbation frequency, or pulmonary rehabilitation participation between subgroups.

**Table 2:** Baseline study cohort characteristics by subgroup. There were 25 participants in the incident low muscle mass phenotype and 221 participants in the stable muscle mass phenotype based on final study visit FFMI and/or ASMI data. Values are listed as mean with standard deviation, unless otherwise specified by *, indicating median with interquartile range

	SM Subgroup (*n* = 221)	LM Subgroup (*n* = 25)	P-Value
Age (y)	64.7 (5.8)	63.9 (5.3)	NS
Male sex (n, %)	114 (52%)	15 (60%)	NS
Caucasian race (n, %)	205 (93%)	25 (100%)	NS
Active smoker (n, %)	84 (38%)	15 (60%)	0.04
Pack years *	45 (30–60)	42 (35–70)	NS
Inhaled corticosteroids (n, %)	23 (10%)	0 (0%)	NS
Oral corticosteroids (n, %)	3 (1%)	1 (4%)	NS
BMI (kg/m^2^)	29.4 (3.4)	25.7 (2.0)	< 0.0001
FFMI (kg/m^2^)	19.7 (2.9)	17.8 (1.7)	0.002
ASMI (kg/m^2^)	8.2 (1.2)	7.3 (0.9)	0.0003
Total body fat mass (kg)	28.1 (7.7)	22.9 (4.7)	0.001
Spirometry			
FEV_1_/FVC FEV_1_ predicted (%) DLco predicted (%)	0.70 (0.11) 88.1 (18.5) 78.3 (16.5)	0.66 (0.11) 84.6 (20.5) 73.3 (12.3)	NS NS NS
COPD (n, %) GOLD I (n, %) GOLD II (n, %) GOLD III (n %) GOLD IV (n, %)	89 (40%) 37 (42%) 44 (49%) 8 (9%) 0 (0%)	13 (60%) 5 (38%) 7 (54%) 1 (8%) 0 (0%)	NS
Emphysema by EScore (n, %) Trace/Minimal (n, %) Mild (n, %) Moderate (n, %) Severe (n, %) Very Severe (n, %)	109 (49%) 62 (57%) 20 (18%) 21 (19%) 4 (4%) 2 (2%)	14 (56%) 9 (64%) 4 (29%) 0 (0%) 0 (0%) 1 (7%)	NS
Perc15 (HU)	-902 (25)	-903 (21)	NS
SGRQ Total score *	12 (6–27)	9 (5–30)	NS
SGRQ Activity score *	19 (6–42)	17 (6–42)	NS
SGRQ Impacts score *	5 (0–13)	0 (0–17)	NS
SGRQ Symptoms score *	23 (10–38)	22 (10–30)	NS
mMRC score *	1 (0–1)	0 (0–1)	NS
Walk distance (m)	454 (143)	479 (149)	NS
Severe exacerbation (n, %)	9 (4%)	1 (4%)	NS
Pulmonary rehabilitation (n, %)	2 (< 1%)	0 (0%)	NS

**Fig. 4:**
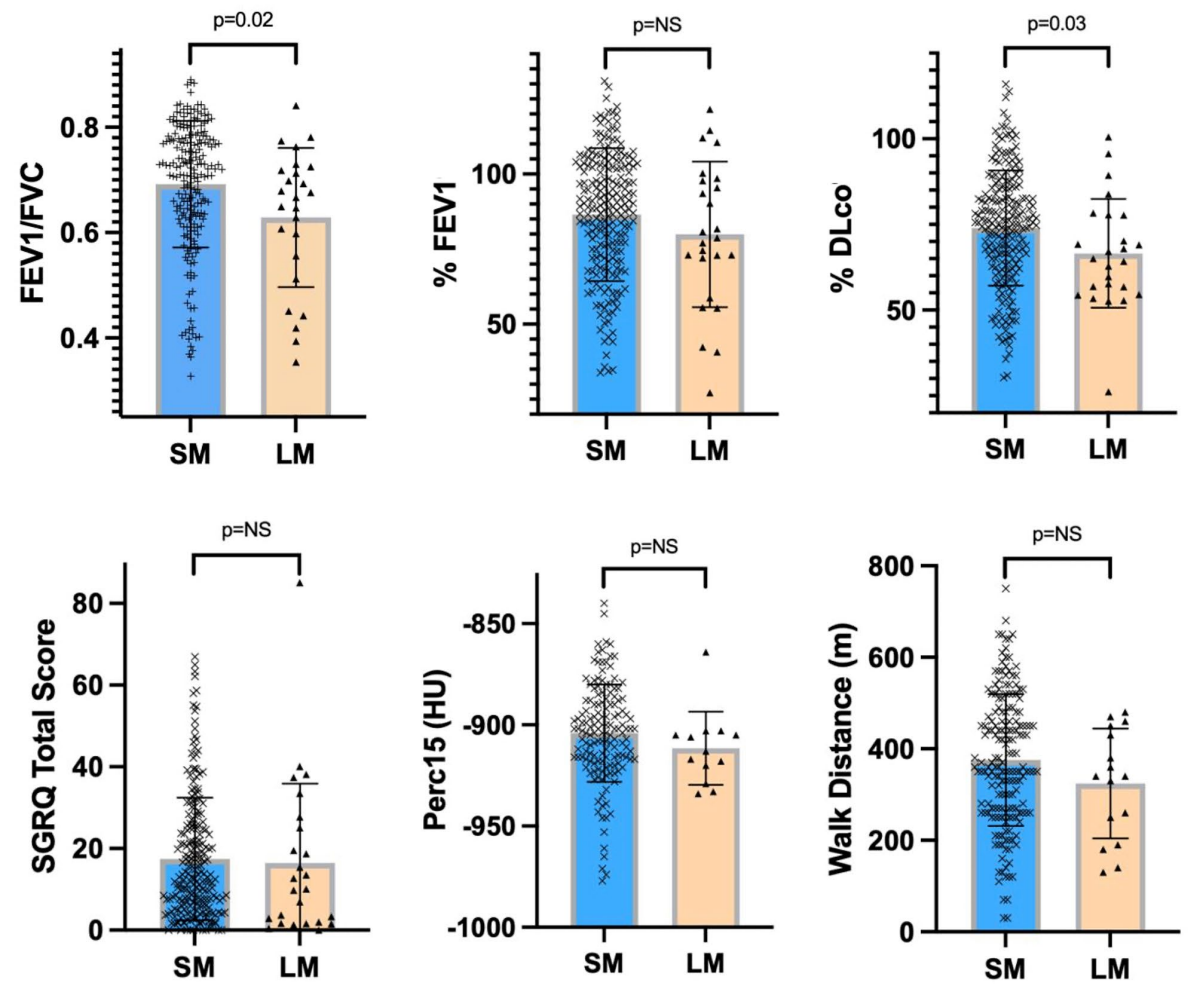
LM subgroup demonstrated lower lung function at final follow-up compared with SM subgroup. Bar graph with associated dot plot representation by SM and LM subgroup of FEV_1_/FVC, %FEV_1_, %DLco, SGRQ Total score, Perc15, and walk distance, with associated p-value

We compared key final follow-up study cohort characteristics between the LM and SM subgroups (Fig. 4). In addition to lower anthropometric measures and greater active tobacco use, participants in the LM subgroup had greater prevalence of COPD (68% v. 43%, *p* = 0.02), lower FEV_1_/FVC (0.63 ± 0.12 v. 0.69 ± 0.12, *p* = 0.02), and lower DLco% (66.5 ± 15.9% v. 73.9 ± 16.8%, *p* = 0.03). There were trends toward lower %FEV_1_, greater Perc15-measured emphysema burden, and lower walk distance (Supplemental Table 1). There were no differences in age, gender distribution, total pack years, respiratory symptoms, severe exacerbation frequency, pulmonary rehabilitation participation, or mortality between subgroups.

**Fig. 5:**
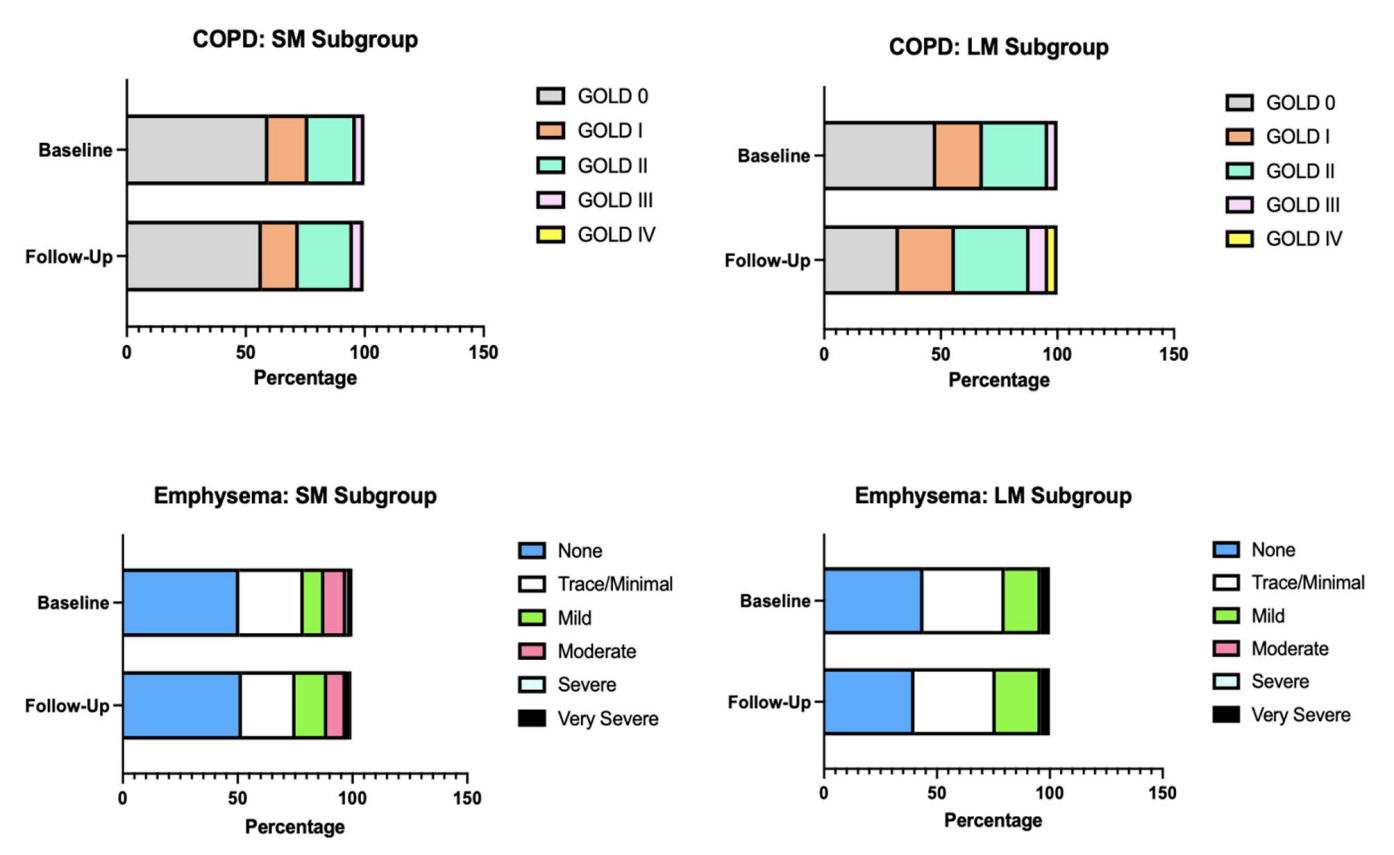
LM subgroup demonstrated greater rates of airflow obstruction and emphysema progression over time compared to the SM subgroup. COPD grouping was defined using GOLD classification. Emphysema grouping was defined using Semi-quantitative EScore classification

In addition to greater annual rate of FFMI decline over time (Fig. 3), participants in the LM subgroup had greater annual rate of Perc15-measured emphysema progression (-2.1 HU/year v. -0.6 HU/year, *p* = 0.04) compared to participants in the SM subgroup (Supplemental Table 2). The LM subgroup trended toward greater annual rates of ASMI and %FEV_1_ decline. Despite significant differences in annual rates of FFMI change between subgroups, there was no difference in annual rates of BMI change. There was no difference in annual rate of walk distance change. During the time interval between baseline to final follow-up, the LM subgroup had greater rates of %FEV_1_ and emphysema progression compared to the SM subgroup (Fig. 5).

### Muscle loss is associated with lower circulating leptin

Compared with the SM subgroup, the LM subgroup had lower circulating leptin levels at baseline (14.9 ± 10.1 ng/mL v. 24.4 ± 20.9 ng/mL, *p* = 0.03) and at follow-up (10.9 ± 6.1 ng/mL v. 23.5 ± 22.1 ng/mL, *p* = 0.001) (Table 3). Adiponectin and resistin levels were similar between groups. In multivariable logistic regression modeling controlling for age, gender, %FEV_1_, active smoking status, corticosteroid use, and duration of follow-up, the odds of incident low muscle mass was lower for every unit of plasma leptin increase at baseline (OR 0.96, 95% CI 0.92–0.99, *p* = 0.047) and at follow-up (OR 0.88, 95% CI 0.81–0.95, *p* = 0.002) (Table 4). The optimal baseline plasma leptin cut-point for the discrimination of incident muscle loss was 27.2 ng/mL (Supplementary Fig. 1).

**Table 3:** Plasma adipokines by subgroup. Plasma levels of adiponectin and leptin were measured at the baseline and final follow-up study visit. Values are listed as mean with standard deviation, p-values are listed for comparisons between muscle subgroup

	SM Subgroup (*n* = 221)	LM Subgroup (*n* = 25)	P-Value
**Baseline**			
Adiponectin (ng/mL)	6736 (3054)	6325 (2377)	NS
Leptin (ng/mL)	24.0 (20.9)	14.9 (10.1)	0.04
Resistin (ng/mL)	6.1 (2.1)	6.0 (2.1)	NS
**Final Follow-Up**			
Adiponectin (ng/mL)	6742 (3307)	6541 (2597)	NS
Leptin (ng/mL)	23.5 (22.1)	10.9 (6.1)	0.001
Resistin (ng/mL)	6.7 (2.5)	6.3 (2.5)	NS

**Table 4:** Multivariable regression of plasma adipokines by subgroup. Multivariable logistic regression modeling was used to evaluate baseline and follow-up plasma leptin levels and incident low muscle mass, with results shown as odds ratio (OR) and 95% confidence interval with associated p-value. Multivariable partial correlation coefficient modeling was used to evaluate baseline and follow-up leptin levels and DXA-derived total body fat mass (kg), with results shown as partial Pearson correlation coefficient (*r*) with associated p-value. Covariates include age, gender, %FEV_1_, active smoking status, corticosteroid use, duration of follow-up

	OR (95% CI)	P-Value	Partial *r*	P-Value
Baseline leptin (ng/mL)	0.96 (0.92–0.99)	0.047	0.58	< 0.0001
Follow-up leptin (ng/mL)	0.88 (0.81–0.95)	0.002	0.59	< 0.0001

## Discussion

We showed that tobacco-exposed individuals with normal baseline muscle mass who progress to low muscle mass have, at baseline, higher rates of active tobacco use and lower circulating leptin levels, but are similar in age, gender distribution, and lung disease status compared with tobacco-exposed individuals who maintain or gain muscle mass over time. Incident low muscle mass is associated with lower lung function, emphysema progression, and the persistence of low circulating leptin levels at follow-up. A higher annual rate of FFMI decline in the low muscle mass subgroup was not reflected by a higher annual rate of BMI decline. These findings suggest that muscle loss and lung disease progression are synchronous processes and reaffirms the importance of comprehensive musculoskeletal assessment in conjunction with lung disease assessment over time in individuals with tobacco exposure.

Our findings describing clinical characteristics associated with progression to incident low muscle mass using gold standard DXA measurements have important clinical implications. While prior studies report cross-sectional and longitudinal associations between FFMI-derived muscle loss and airflow obstruction, emphysema, and mortality, [19–21, 41] these studies include cohorts in which significant proportions of participants have prevalent low muscle mass or sarcopenia. Therefore, we currently have limited insight into the natural history and progression to early musculoskeletal disease in tobacco-related lung disease, as well as the risk factors that contribute to the development of low muscle mass over time. Importantly, while BMI has traditionally been used for risk stratification in COPD, this anthropometric measure may not accurately reflect true muscle mass [14, 42]. This is consistent with our results demonstrating significant differences in annual rates of FFMI change, but no differences in annual rates of BMI change, between the LM and SM subgroups over time. As such, our study highlights the importance of closely monitoring muscle mass using gold standard DXA measurements, or more precise techniques that differentiate skeletal muscle from adipose tissue, prior to the development and progression of sarcopenia and frailty in tobacco-exposed individuals to allow for early targeted therapies such as pulmonary rehabilitation. While measures of muscle strength were not readily available for this study, it is an important component of sarcopenia criteria that may occur prior to muscle loss. Therefore, early evaluation and interventions should also incorporate muscle strength testing.

We found that circulating leptin levels were lower at baseline and at follow-up in participants with incident low muscle mass compared to participants with stable muscle mass over time. We also discovered a positive correlation (adjusted partial Pearson *r* 0.59, *p* < 0.0001) between circulating leptin levels and adipose tissue content using DXA-derived total body fat content in our tobacco-exposed cohort. We did not find differences in circulating adiponectin or resistin levels between subgroups. Leptin is a circulating adipokine central to energy homeostasis and inflammatory response regulation [43]. Prior studies have shown that individuals with COPD have lower serum leptin levels compared with healthy controls, [44] and that age-related reductions in muscle mass and bone strength are associated with reduced leptin levels [45]. The critical role of leptin in regulating muscle mass and function is also well-described in animal models, where leptin loss-of-function (*ob*/*ob*) or lipodystrophic fat-free mice had lower muscle fiber size and reduced peak contractile muscle strength, both of which were partially rescued with leptin administration or reconstitution of 10% normal adipose tissue, respectively [46–48]. In concordance with these studies, our findings suggest that leptin plays a key role in adipose-muscle signaling and is involved in mechanisms of muscle loss in individuals with tobacco exposure. Due to the emergence of leptin replacement therapy, which has primarily been utilized for the treatment of lipodystrophy, [49] future studies targeting this adipokine pathway in individuals with reduced muscle loss may be an important area of research.

We demonstrated different patterns of muscle mass change in a tobacco-exposed cohort with normal baseline muscle mass at cohort entry. We found no differences in lung function, emphysema, or respiratory symptoms between the LM and SM subgroups at baseline, but significantly higher prevalence of COPD and greater lung disease burden in the LM subgroup at follow-up. Whether this is the result of the “spill-over” inflammation phenomenon or concomitant systemic inflammation affecting both lung and skeletal muscle, [50] our data suggests that muscle loss and lung disease progression are synchronous processes, rather than musculoskeletal dysfunction due to lung disease. This is congruent with a recent COPDGene study demonstrating that lung disease severity did not significantly correlate with musculoskeletal comorbidity burden: compared with tobacco-exposed individuals with COPD, tobacco-exposed individuals with preserved ratio-impaired spirometry (PRISm) had stronger correlations with measures of muscle weakness [51]. However, it is also possible that the difference in the follow-up period may have affected the incidence of COPD onset between the two subgroups. Our study emphasizes the importance of comprehensive and serial monitoring of musculoskeletal assessments, in conjunction with lung disease assessments, over time in this high-risk population.

There are several strengths of our study worth highlighting. First, we used gold standard DXA measures of body composition to evaluate changes in muscle mass in our tobacco-exposed cohort, which allowed us to define low muscle mass using standard, clinically accepted definitions. Second, our cohort included tobacco-exposed individuals without airflow obstruction (58%), which differentiates it from prior studies of musculoskeletal dysfunction that focused primarily on tobacco-exposed individuals with spirometrically-defined COPD. The inclusion of individuals with tobacco exposure and preserved lung function is critical given the growing body of evidence demonstrating increased respiratory lung disease morbidity and mortality in tobacco-exposed individuals without airflow obstruction [28, 52]. Third, this study allowed us to make important inferences about muscle loss and lung disease progression over time.

There are several limitations of this study. First, this cohort is comprised of participants with mild to moderate airflow obstruction. Therefore, our results may not be generalizable to individuals with severe obstruction, and we cannot make inferences about the impact of severe respiratory exacerbations on the development of low muscle mass, which has been shown in prior studies [53]. Second, we only have data for tobacco-exposed individuals who are 64 years of age or older and excluded those who already had low muscle mass at cohort entry. As a result, it is possible that we did not capture a third subgroup of tobacco-exposed individuals, those with lower muscle mass earlier in life and slow decline over time who develop low muscle mass, analogous to the lung function decline trajectory described by Lange and colleagues, where the subgroup with low %FEV_1_ at cohort inception and slow %FEV_1_ decline developed COPD over time [54]. Third, our predominantly Caucasian study population reflected the demographics of western Pennsylvania, eastern Ohio, and West Virginia, but may not have adequately captured global minority or racial differences in tobacco-exposed individuals with and without COPD. Fourth, due to the small percentage of participants who underwent pulmonary rehabilitation (< 1% at baseline, 3% at final follow-up), we did not have consistent data on the effects of this physical intervention on skeletal muscle mass.

In conclusion, we identified a subgroup of tobacco-exposed individuals with normal baseline muscle mass who progressed to incident low muscle mass. Over time, individuals in this subgroup demonstrated greater active smoking prevalence, more airflow obstruction, greater emphysema progression, and lower circulating leptin compared to individuals with stable muscle mass. This study demonstrates that importance of following musculoskeletal comorbidities over time in high-risk individuals and suggests possible mechanisms involved in tobacco-related muscle loss.

### Electronic supplementary material

Below is the link to the electronic supplementary material.

Supplementary Material 1

## Data Availability

The datasets generated and/or analyzed during the current study are available from the corresponding author on reasonable request.

## Abbreviations

ASMI: Appendicular skeletal mass index
ATS: American Thoracic Society
BMI: Body mass index
COPD: Chronic obstructive pulmonary disease
CT: Computed tomography
DLco: Diffusing capacity of lungs for carbon monoxide
DXA: Dual energy x-ray absorptiometry
EScore: Emphysema Score
FEV_1_: Forced expiratory volume in 1 s
FFMI: Fat free mass index
FVC: Forced vital capacity
GOLD: Gold Initiative for Chronic Obstructive Lung Disease
ISWT: Incremental shuttle walk test
LM: Incident low muscle mass subgroup
mMRC: Modified Medical Research Council dyspnea score
Perc15: 15th percentile voxel point
SCCOR: Specialized Centers of Clinically Oriented Research
SGRQ: St. George’s Respiratory Questionnaire
SM: Stable muscle mass subgroup
