# Supplementary material for: Incident low muscle mass is associated with greater lung disease and lower circulating leptin in a tobacco-exposed longitudinal cohort
Source: Respir Res. 2023 Sep 22;24:224. doi: 10.1186/s12931-023-02521-3 (PMC10515430; doi:10.1186/s12931-023-02521-3)
Supplement: Supplementary file 1 — Supplementary Material 1 [file 12931_2023_2521_MOESM1_ESM.docx]

|  | **SM Subgroup**  **(*n*=221)** | **LM Subgroup**  **(*n*=25)** | **P-Value** |
| --- | --- | --- | --- |
| **Age (y)** | 70.6 (5.8) | 71.4 (5.7) | NS |
| **Male sex (n, %)** | 114 (52%) | 15 (60%) | NS |
| **Caucasian race (n, %)** | 205 (93%) | 25 (100%) | NS |
| **Active smoker (n, %)** | 66 (30%) | 14 (56%) | 0.01 |
| **Inhaled corticosteroids (n, %)** | 43 (19%) | 1 (4%) | NS |
| **Oral corticosteroids (n, %)** | 5 (2%) | 1 (4%) | NS |
| **BMI (kg/m^2^)** | 29.4 (4.0) | 24.7 (2.2) | <0.0001 |
| **FFMI (kg/m^2^)** | 19.5 (2.4) | 16.7 (1.6) | <0.0001 |
| **ASMI (kg/m^2^)** | 8.0 (1.2) | 6.7 (0.9) | <0.0001 |
| **Total body fat mass (kg)** | 28.6 (8.2) | 21.6 (5.1) | <0.0001 |
| **Spirometry**  **FEV_1_/FVC**  **FEV_1_ predicted (%)**  **DLco predicted (%)** | 0.69 (0.12)  89.0 (20.6)  73.9 (16.8) | 0.63 (0.12)  78.8 (24.2)  66.5 (15.9) | 0.02  0.05  0.03 |
| **COPD (n, %)**  **GOLD I (n, %)**  **GOLD II (n, %)**  **GOLD III (n %)**  **GOLD IV (n, %)** | 94 (43%)  34 (36%)  50 (53%)  10 (11%)  0 (0%) | 17 (68%)  6 (35%)  8 (47%)  2 (12%)  1 (6%) | 0.02 |
| **Emphysema by EScore (n, %)**  **Trace/Minimal (n, %)**  **Mild (n, %)**  **Moderate (n, %)**  **Severe (n, %)**  **Very Severe (n, %)** | 101 (55%)  49 (58%)  29 (20%)  18 (17%)  3 (3%)  2 (2%) | 15 (63%)  9 (60%)  5 (33%)  0 (0%)  0 (0%)  1 (7%) | NS |
| **Perc15 (HU)** | -904 (24) | -912 (18) | 0.09 |
| **SGRQ Total score *** | 13 (6-25) | 10 (2-25) | NS |
| **SGRQ Activity score *** | 23 (6-42) | 23 (0-41) | NS |
| **SGRQ Impacts score *** | 5 (0-13) | 2 (0-12) | NS |
| **SGRQ Symptoms score *** | 22 (9-35) | 11 (8-23) | NS |
| **mMRC score *** | 1 (0-1) | 0 (0-1) | NS |
| **Walk distance (m)** | 376 (144) | 324 (120) | 0.08 |
| **Severe exacerbation (n, %)** | 7 (3%) | 2 (8%) | NS |
| **Pulmonary rehabilitation (n, %)** | 7 (3%) | 1 (4%) | NS |
| **Deceased (n, %)** | 15 (7%) | 3 (12%) | NS |

**Supplemental Table 1: Final follow-up study cohort characteristics by subgroup.** A total of 246 participants had demographic, clinical, radiographic, and body composition data. Values are listed as mean with standard deviation, unless otherwise specified by *, indicating median with interquartile range.

|  | **SM Subgroup**  **(*n*=221)** | **LM Subgroup**  **(*n*=25)** | **P-Value** |
| --- | --- | --- | --- |
| **Δ BMI (kg/m^2^)** | -0.11 (0.47) | -0.13 (0.29) | NS |
| **Δ FFMI (kg/m^2^)** | -0.04 (0.23) | -0.17 (0.1) | 0.006 |
| **Δ ASMI (kg/m^2^)** | -0.04 (0.12) | -0.08 (0.04) | 0.09 |
| **Δ Total body fat mass (kg)** | 0.07 (0.7) | -0.2 (0.6) | NS |
| **Spirometry**  **Δ FEV_1_/FVC**  **Δ FEV_1_ predicted (%)**  **Δ DLco predicted (%)** | -0.002 (0.009)  -0.18 (2.0)  -0.70 (2.7) | -0.004 (0.008)  -0.85 (1.2)  -1.2 (2.0) | NS  0.10  NS |
| **Δ Perc15 (HU)** | -0.62 (3.5) | -2.1 (1.7) | 0.04 |
| **Δ SGRQ Total score *** | 0.04 (2.1) | -0.31 (0.9) | NS |
| **Δ SGRQ Activity score *** | 0.07 (3.6) | -0.33 (1.8) | NS |
| **Δ SGRQ Impacts score *** | 0.12 (2.0) | -0.13 (1.1) | NS |
| **Δ SGRQ Symptoms score *** | -0.22 (3.9) | -0.64 (3.2) | NS |
| **Δ mMRC score *** | 0.03 (0.18) | 0.02 (0.14) | NS |
| **Δ Walk distance (m)** | -14 (26) | -20 (20) | NS |
| **Δ Adiponectin (ng/mL)** | -12 (360) | 55 (284) | NS |
| **Δ Leptin (ng/mL)** | -0.25 (2.1) | -0.50 (1.2) | NS |
| **Δ Resistin (ng/mL)** | 0.59 (1.6) | 0.36 (1.1) | NS |

**Supplemental Table 2: Annual change in study cohort characteristics by subgroup.** A total of 246 participants had demographic, clinical, radiographic, and body composition data. Values are listed as mean with standard deviation, unless otherwise specified by *, indicating median with interquartile range.

**Supplemental Figure 1: Receiver operator curve for baseline plasma leptin level and incident low muscle mass.** The AUC (area under ROC curve) is 0.69 with an optimal baseline plasma leptin cut-point of 27.2 ng/mL by Youden index. Covariates include age, gender, %FEV_1_, active smoking status, corticosteroid use, duration of follow-up.
